# Supplementary material for: 21st-century modeled permafrost carbon emissions accelerated by abrupt thaw beneath lakes
Source: Nat Commun. 2018 Aug 15;9:3262. doi: 10.1038/s41467-018-05738-9 (PMC6093858; doi:10.1038/s41467-018-05738-9)
Supplement: Supplementary file 1 — Supplementary Information [file 41467_2018_5738_MOESM1_ESM.pdf]

Supplementary Information

21st-century modeled permafrost carbon emissions accelerated by abrupt thaw beneath lakes

Walter Anthony et al.

Supplementary Figures

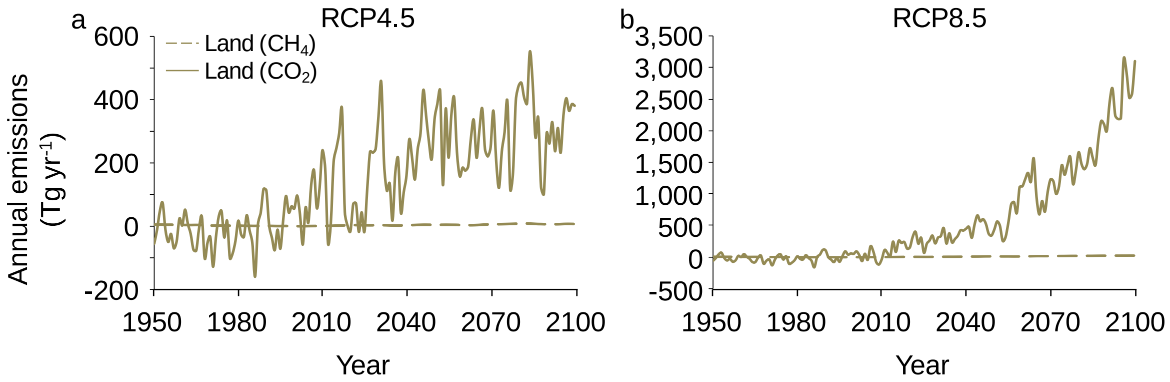

Supplementary Figure 1. CLM4.5BGC methane (CH_4) and carbon dioxide (CO_2) emissions from land in the circumpolar permafrost region. Fluxes according to RCP4.5 (a) and RCP8.5 (b) are shown on different y-axis scales. Negative CO_2 values that occur in some years during 1950 to 2026 represent times when atmospheric uptake by plants is stimulated more than decomposition of soil organic matter.

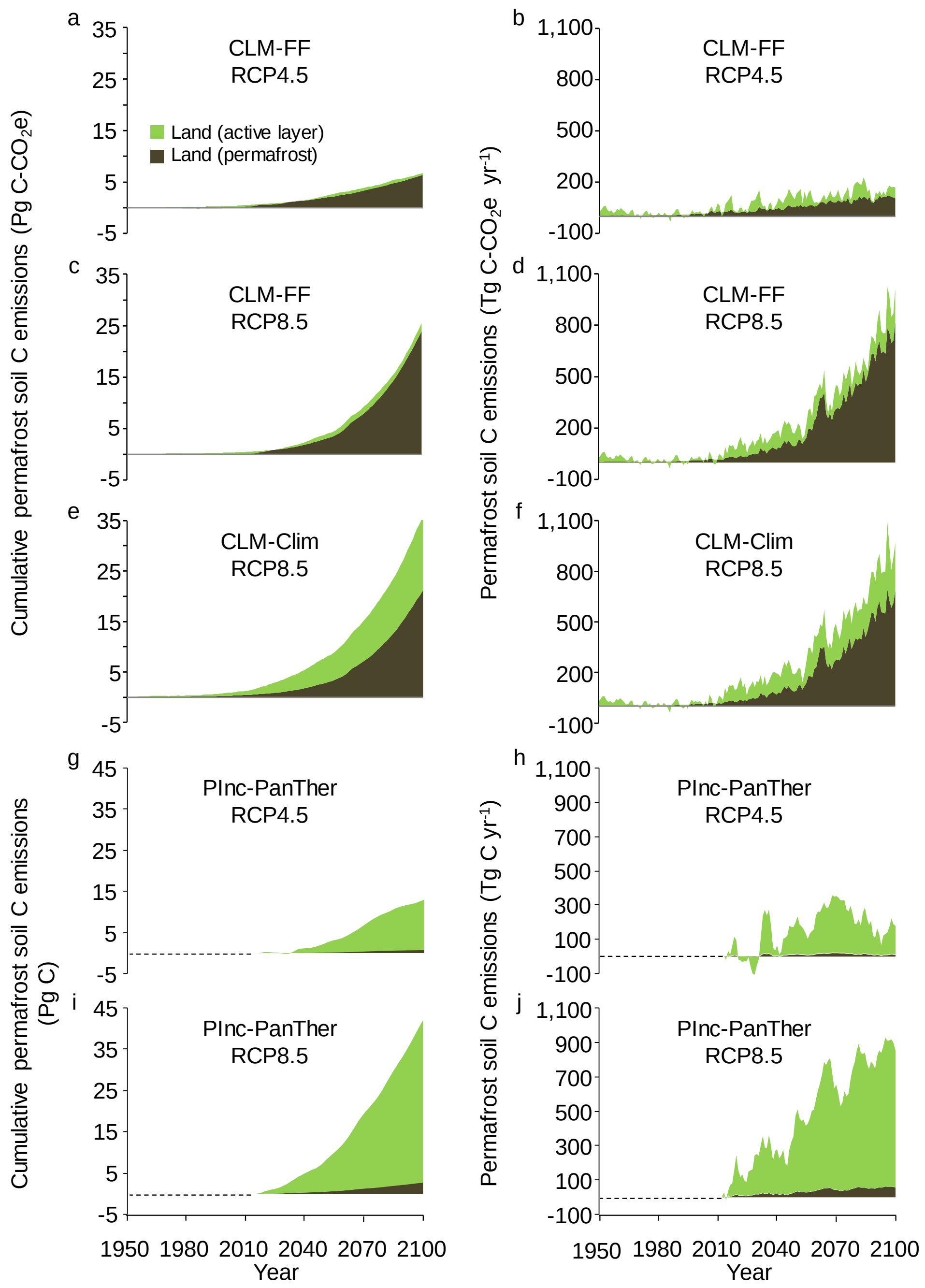

Supplementary Figure 2. Pan-arctic permafrost carbon emissions from gradual thaw (land) during 1950 to 2100. Permafrost soil carbon emissions, modeled according to representative concentration pathway (RCP) 4.5 and RCP8.5 scenarios, are distinguished for warming-enhanced decomposition of active layer soil carbon above present-day permafrost (green) and gradual thaw of what is initially permafrost (brown) from the Community Land Model, version 4.5 (CLM4.5BGC) fully-forced scenario[1] (CLM-FF, panels a-d) and the climatically-forced scenario[1] (CLM-Clim, panels e-f). In CLM-FF future elevated atmospheric CO_2 concentration stimulating net primary production is accounted for; CLM-Clim (RCP8.5 only) lacks this CO_2 fertilization effect. The simplified, data-constrained Permafrost Carbon Network Incubation–Panarctic Thermal model (PInc-PanTher, panels g-j) attributed nearly all (94%) of cumulative terrestrial permafrost-region soil emissions during 2010 to 2100 to active-layer soil decomposition, with gradual, top-down thaw of permafrost contributing only ~6% (2). Based on this ratio, we assumed a constant 94% active layer source (~6% permafrost thaw source) throughout the century, although Koven et al. (2) does not provide information on the ratio's temporal variability. The dashed line in panels g-j indicates the period of unavailable PInc-PanTher data. Note different units for CLM (C-CO_2e) versus PInc-PanTher (C) model emissions.

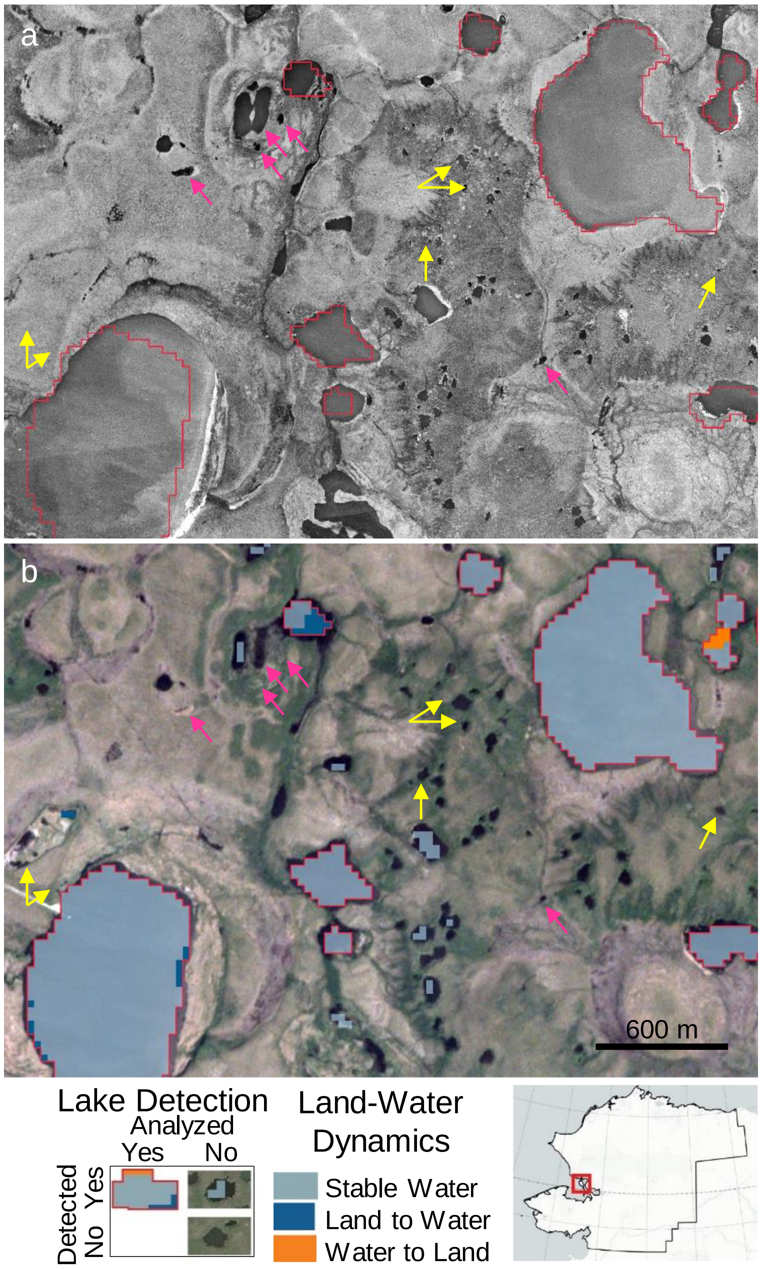

Supplementary Figure 3. Magnified view of 1999-2014 lake area loss and gain map results for the Baldwin Peninsula, northwest Alaska. (a) 1952 aerial photograph (Mosaicked Aerial Photo Single Frames); (b) Background image: PlanetScope image acquired on July 3, 2017. Red lines indicate the maximum lake extent detected in the 30-m Landsat analysis. Yellow arrows show select examples of small lakes that formed or expanded since 1952, which were not detected in the 30-m Landsat analysis or not analyzed due to minimum mapping unit constraints. Magenta arrows show select examples of undetected lake area loss since 1952.

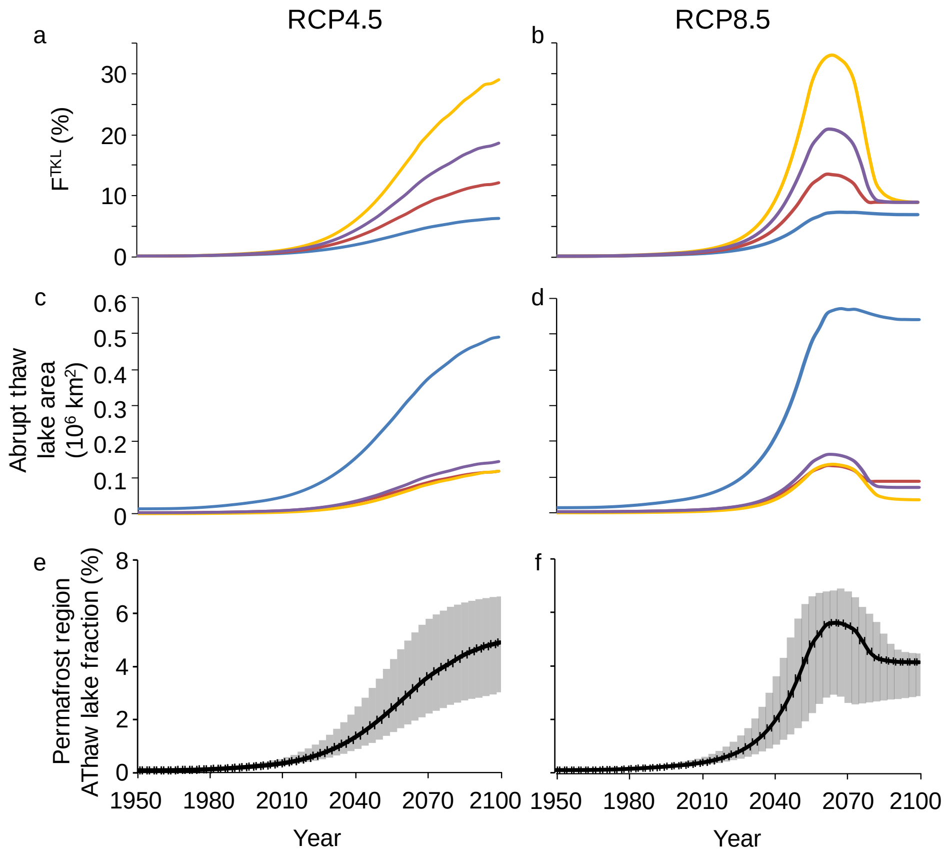

g

Soil class	RCP4.5 F^{TKLmax} median	RCP4.5 F^{TKLmax} (68% range)	RCP8.5 F^{TKLmax} median	RCP8.5 F^{TKLmax} (68% range)
MS	6%	(4% - 8%)	7%	(4% - 9%)
ORG	12%	(7% - 16%)	14%	(10% - 17%)
YED	29%	(16% - 41%)	33%	(23% - 42%)
R_{TKL}	19%	(11% - 26%)	21%	(15% - 27%)

Supplementary Figure 4. AThaw-modeled extent of abrupt thaw during 1950 to 2100 for Representative Concentration Pathways (RCP) 4.5 and 8.5. (a, b) The net thermokarst lake fraction F_{TKL} of the landscape representing the net extent of abrupt thaw by new thermokarst lake formation and drainage in ice-rich permafrost-dominated soils according to soil type: mineral (MS—), organic (ORG—), Yedoma (YED—) and Refrozen thermokarst deposits (R_{TKL}—). (c, d) Abrupt thaw net lake area within four soil types. (e, f) Percent of the permafrost region terrestrial landscape (17.8 million km^2) occupied by abrupt thaw lakes. (g) Maximum thermokarst lake fraction F^{TKLmax} according to soil type. Median values are shown in *a*-*d*; median and the 68% uncertainty range for are shown in *e*-*g*. In *b*, a rapid drop in thermokarst lake area occurs after 2060 caused by warming-induced lake drainage in the strong warming scenario RCP8.5. Values in *a* and *b* are higher than *e* and *f* because not all permafrost-region soils (17.8 million km^2) (3) are vulnerable to thermokarst, particularly soils lacking high ice contents, and because panels *a* and *b* refer only to those four classes of ice-rich soils susceptible to thermokarst (~10 million km^2; Supplementary Table 1).

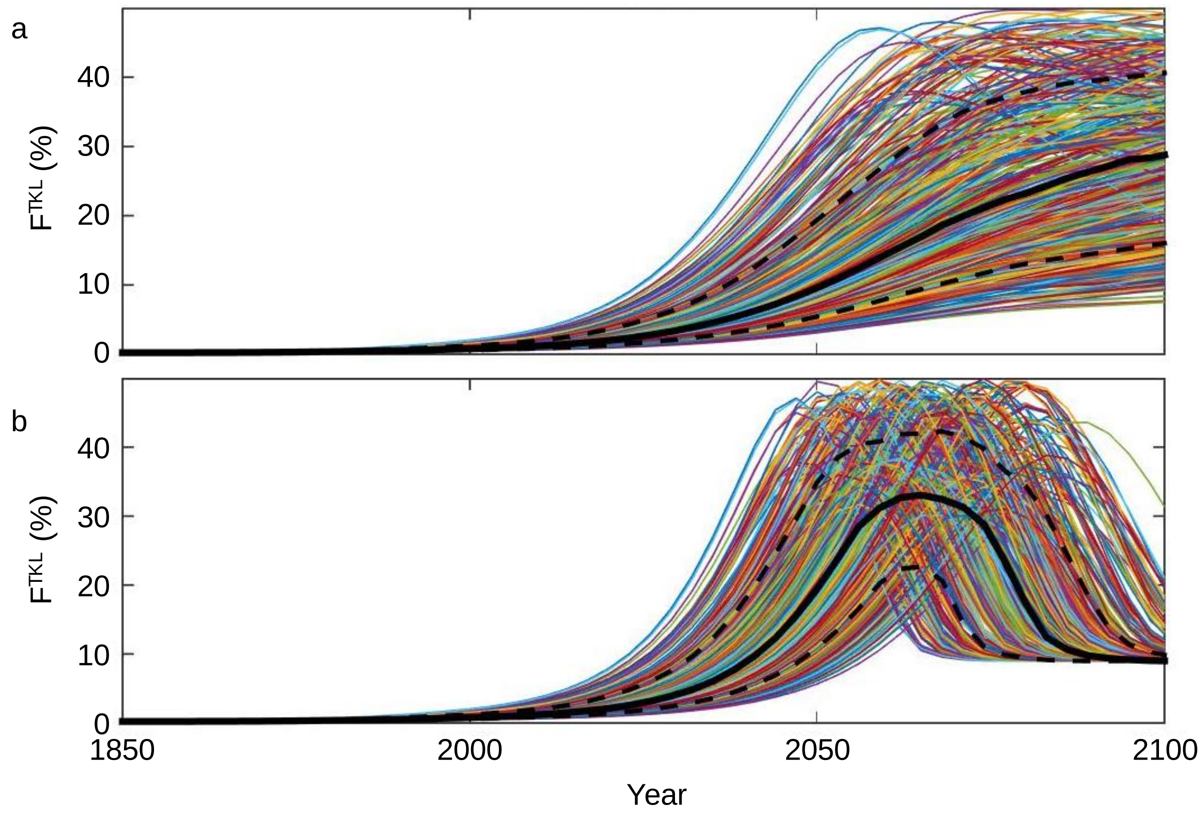

Supplementary Figure 5. Ensemble spread of simulated trajectories of thermokarst lake formation and drainage in the Yedoma soil class for RCP4.5 (a) and RCP8.5 (b). Data show the net extent to which the yedoma landscape is covered by newly formed lakes (in percent). Black lines denote the ensemble median, dashed line the 68% uncertainty range. A total of 18 model parameters were varied simultaneously; however, sampling of two specific lake parameters (F^{TKLmax} and dT'^{TKLmax}) generate most of the spread in lake dynamics shown here. The spread mimics differing realizations of lake formation and drainage. While net lake area is lower for other soil classes (mineral, organic and refrozen thermokarst deposits) (Methods, Supplementary Fig. 4, Supplementary Table 1), the temporal dynamics of F^{TKL}, governed by dT'^{TKLmax}, are similar (data not shown).

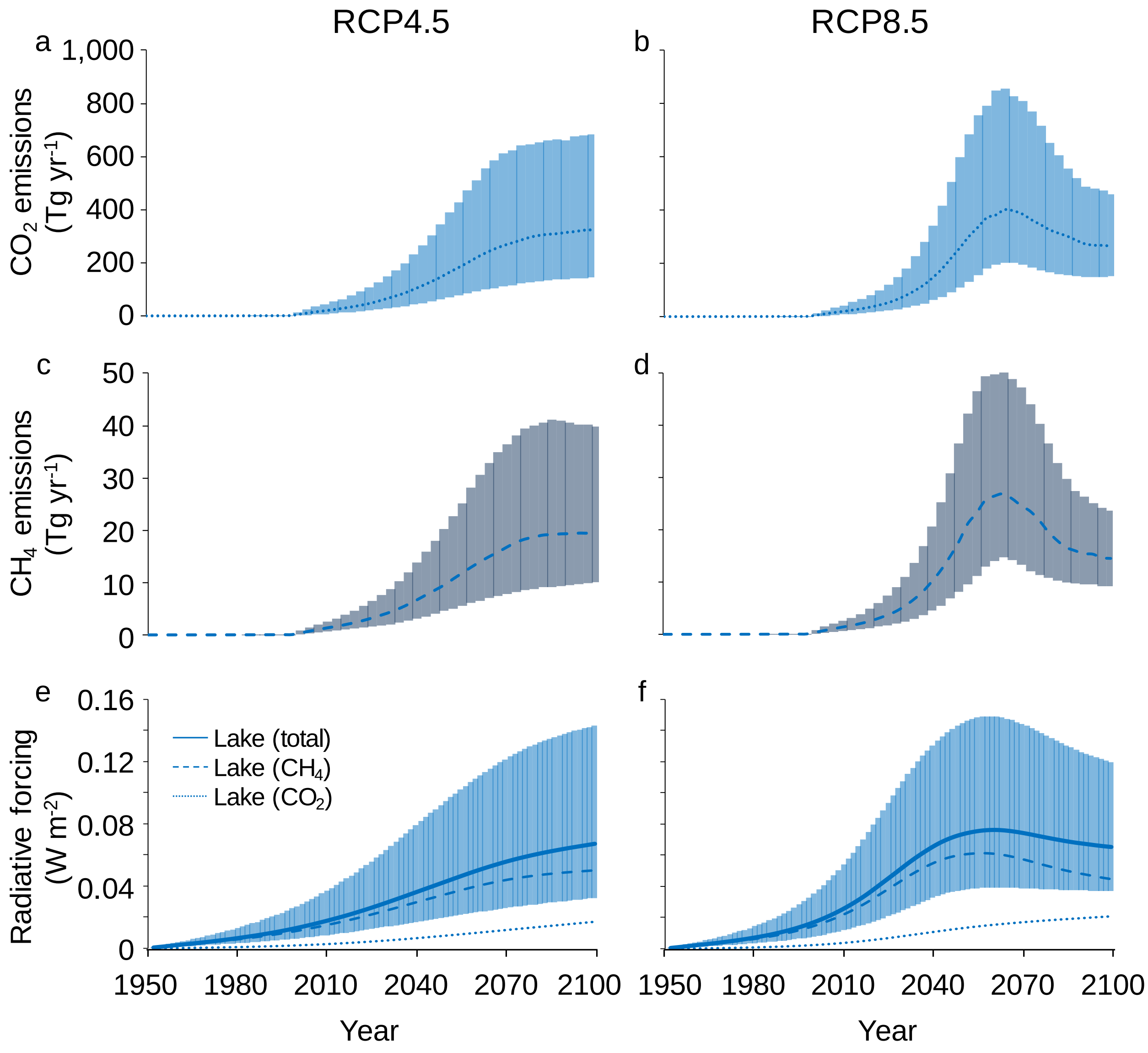

Supplementary Figure 6. Methane and carbon dioxide emissions and associated radiative forcing from newly formed thermokarst lakes according to AThaw. CO_2 (a, b) and CH_4 (c, d) emissions and the associated radiative forcing (e, f) represent the median and 68% uncertainty range from a 500-member AThaw model ensemble, which considers uncertainty in 18 key model parameters containing critical factors that influence AThaw modeled emissions following Schneider von Deimling et al. (4).

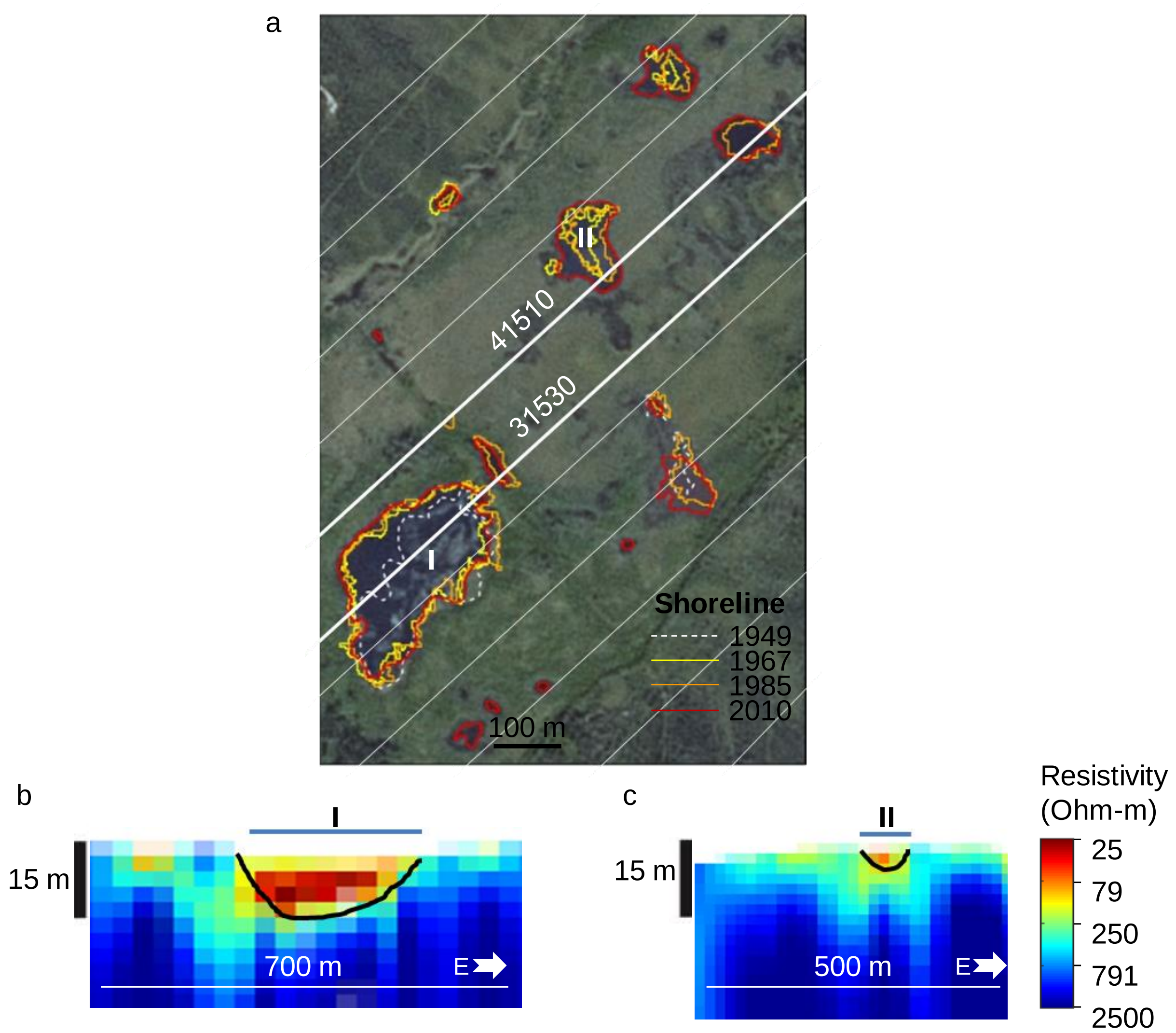

Supplementary Figure 7. EM1DFM resistivity models. (a) Interior Alaska study lakes I and II with electromagnetic survey flight lines shown by white lines on a 2010 SPOT image. (b) Resistivity model from a portion of line 31530 crossing Lake I generated from EM1DFM. (c) Resistivity model from a portion of line 41510 crossing Lake II generated from EM1DFM. Lake locations as solid blue lines. Interpreted thaw depths as solid black lines.

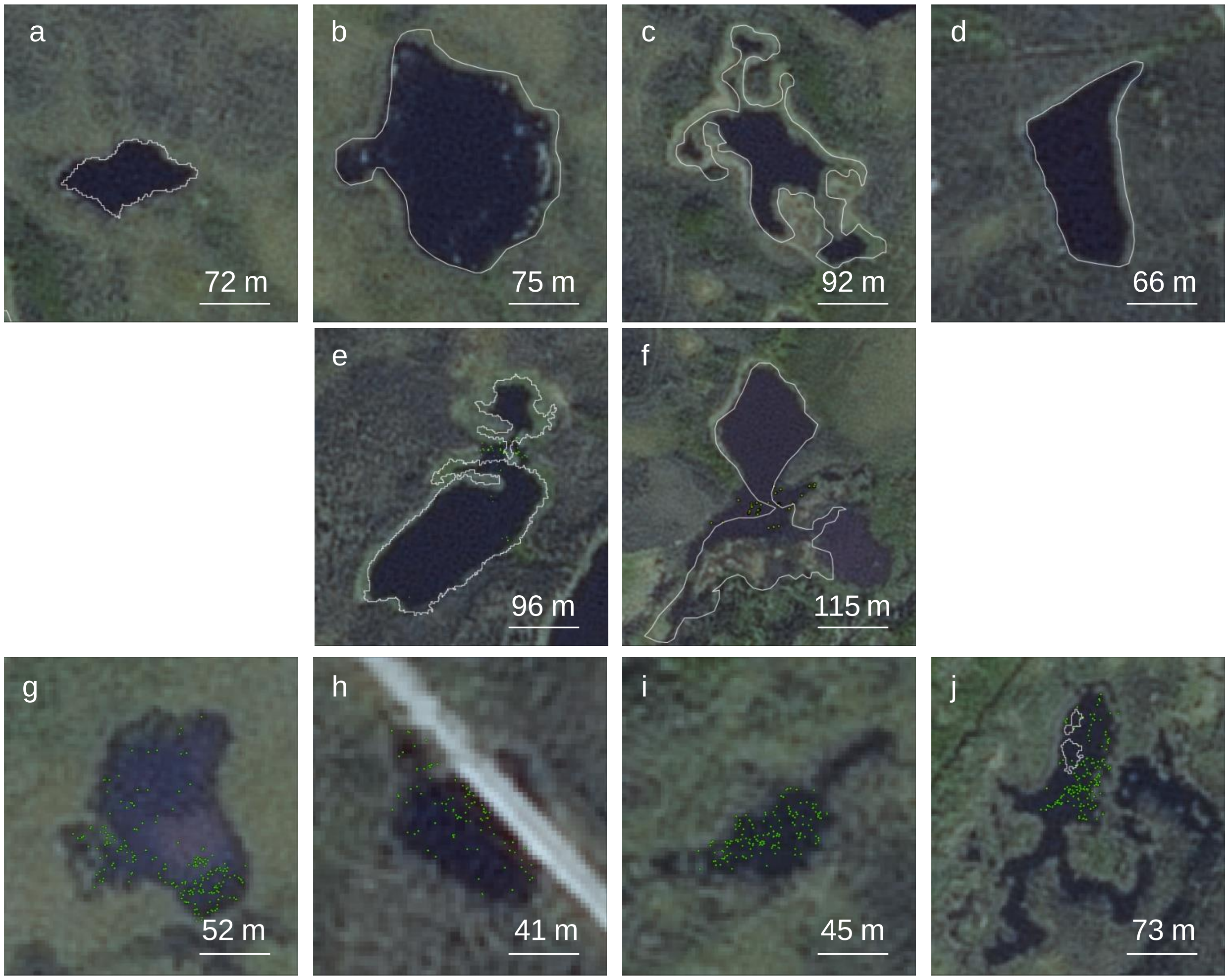

Supplementary Figure 8. Locations of strong hotspot methane (CH_4) seeps in relationship to abrupt thaw. 1949 lake shorelines (white lines) and strong CH_4 seep locations (green dots) are shown on 2010 SPOT images of Goldstream Valley, Alaska thermokarst lakes. In a-d, no large CH_4 seeps were observed in lakes that have not experienced any significant thermokarst expansion since 1949. In e and f, strong CH_4 seeps are largely confined to areas of lakes that have undergone abrupt thaw since 1949. In g-j, strong CH_4 seeps distributed across the surfaces of lakes that were not present on the landscape in 1949. It should be noted that the long, narrow bays in panels i and j were not surveyed for seeps (no data).

Supplementary Tables

Supplementary Table 1. Observation-based AThaw model parameters and uncertainties. Table modified from Schneider von Deimling et al. (4).

Parameter	Unit	Default	Uncertainty range	References
Permafrost carbon inventory				
Mineral soils (MS) 0-3 m (Orthels & Turbels)	Pg C	540	± 40%	Hugelius et al. (3)
Organic soils (ORG) 0-3 m (Histels)	Pg C	120	± 40%	Hugelius et al. (3)
Yedoma (Y) 0-15 m	Pg C	83	± 75%	Strauss et al. (5)
Refrozen thermokarst deposits (RTK)	Pg C			
Surface 0-5 m		128	± 75%	Strauss et al. (5)
Taberal 5-15 m		114	± 75%	Walter Anthony et al. (6)
Fraction fast pool†	%	2.5	1-4	Dutta et al. (7), Burke et al. (8), Schadel et al. (9)
Fraction slow pool	%	45	30-60	Burke et al. (8), Sitch et al. (10), Koven et al. (11)
Carbon release				
Ratio of anaerobic production CH_4: CO_2‡		Fast 1:1	± 20%	Walter Anthony et al. (6)
		Slow 1:7	± 50%	Lee et al. (12)
Q_{10} anaerobic		3	2-6	Walter & Heimann (13)
CH_4 oxidation rate	%	15	10-20	Burke et al. (8)
Permafrost thaw				
Thaw rate (MS aerobic)§	$cm\ yr^{-1}\ K^{-1}$			Frauenfeld et al. (14), Hayes et al. (15), Schaphoff et al. (16)
warm permafrost		1		
cold permafrost		0.1		
Thermal diffusivity scale factor for abrupt thaw lakes‖		9.3	± 30%	Kessler et al. (17)
Thermokarst lake dynamics				
Newly formed thermokarst lake fraction F^{TKLmax}, representing net abrupt-thaw lake formation and drainage	% coverage per latitude	MS 8	± 25%	See Methods
		ORG 16	± 25%	
		Y 40	± 25%	
		RTK 25	± 25%	
High-latitude temperature anomaly dT'^{TKLmax} at F^{TKLmax}¶	°C	5	4-6	See Methods

†For Yedoma deposits, AThaw assumes a two times larger labile fraction (5 ± 3 %) based on rapid sedimentation of organic matter, a condition that favored relatively little decomposition and rapid sequestration of labile organic carbon during syngenetic Yedoma permafrost formation in the late Pleistocene[5]. In contrast, AThaw assumes a reduced labile fraction in taberal sediments of 1% as these deposits were previously thawed beneath lakes over long timescales and are therefore depleted in high-quality organic matter[5,6].

‡Very small ratios of anaerobic CH_4:CO_2 inferred from laboratory incubations were excluded due to the likely large effect of the CO_2 pulse during the initial phase of the incubation.

§Indicated thaw rates are exemplary for warm and cold permafrost (corresponding to a mean annual ground temperature (MAGT) of just below 0 and -10 °C). Thaw rates were calculated by Schneider von Deimling et al. (4) assuming that above-zero temperatures prevail during four months a year and that thaw is driven by a surface temperature warming anomaly of 1 °C.

‖Schneider von Deimling et al. (4) prescribed aggregated thermal diffusivities for soils under aerobic conditions and use scaling factors to determine modified thermal diffusivities under saturated, anaerobic conditions. Scaling factors for thermokarst lakes were tuned to reproduce long-term behavior of talik propagation as simulated by Kessler et al. (17).

¶Early Holocene warming by a few degrees Celsius in Northern Hemisphere land areas[18-20] resulted in rapid and intensive thermokarst activity[6,21-22].

Supplementary Table 2. conversion factors and formulas based on 1 mass unit CH_4 having GWP_{100} of 28 times 1 mass unit CO_2, and where 12, 16, and 44 are the atomic mass of C and molecular masses of CH_4, and CO_2 respectively.

Factor	Formula	Conversions
28		1 mass unit CH_4 = 28 x 1 mass unit CO_2
37.3	28x(16/12) = 37.3	1 mass unit C-CH_4 = 37.3 x 1 mass unit CO_2
7.6	28x(12/44)= 7.6	1 mass unit CH_4 = 7.6 x 1 mass unit C-CO_2
10.2	37.3x(12/44)=10.2	1 mass unit C-CH_4 = 10.2 x 1 mass unit C-CO_2
0.27	(12/44) = 0.27	1 mass unit CO_2 = 0.27 x 1 mass unit C-CO_2

Supplementary Table 3. Gross and net lake area changes and associated carbon fluxes in northern and western Alaska for 1999-2014 determined with Landsat satellite trend analysis. Net lake area change is the sum of gross lake area loss (GLAL) and gross lake area gain (GLAG). The past 60-year normalized gross lake area gain (60-yr normalized GLAG) is also shown, for comparison to other pan-arctic studies summarized in Walter Anthony et al. (23), assuming a constant rate of increase observed during the 15-year period 1999-2014 for the past 60 years. However, AThaw projects more rapid rates of lake formation and expansion driven by higher RCP4.5 and RCP8.5 temperatures from 2020 to 2100 (Supplementary Figs. 4, 5). ΔF_{GLAL} is the change in carbon flux associated with gross lake area loss, calculated as the difference between the flux in the drained thermokarst lake basins (F_{DTLB}) and the flux in the mature lake prior to drainage ($F_{MatureLake}$). ΔF_{GLAG} is the change in carbon flux associated with gross lake area gain, calculated as the difference between the flux in the newly formed thermokarst lake zone (F_{TKzone}) and the flux on land prior to lake formation or expansion (F_{Land}). Flux values for mature lakes, new thermokarst lake areas, drained lake basins and land are shown in Supplementary Table 5 for yedoma and non-yedoma permafrost types.

		Land surface type		
		Yedoma	Non-yedoma	Total
Landscape area	(km^2)	82,954	429,451	512,405
Number of lakes	-	12,342	61,462	73,804
1999 lake area	(km^2)	1,464	11,333	12,798
2014 lake area	(km^2)	1,407	11,214	12,621
Net change	(km^2)	-57	-119	-176
Net change	%	-3.9%	-1.0%	-1.4%
Gross Lake Area Loss (GLAL)	(km^2)	-82	-248	-330
Gross Lake Area Gain (GLAG)	(km^2)	25	129	154
GLAG	%	1.7%	1.1%	1.2%
normalized GLAG (60 yrs)	%	7%	5%	5%
$\Delta F_{GLAL} = F_{DTLB} - F_{MatureLake}$	(kg C-CO_2e km^{-2} yr^{-1})	-254,391	-124,641	
$\Delta F_{GLAG} = F_{TKzone} - F_{Land}$	(kg C-CO_2e km^{-2} yr^{-1})	1,764,287	526,500	
15-yr GLAL change in C flux	(Tg C-CO_2e)	-0.31	-0.46	-0.78
15-yr GLAG change in C flux	(Tg C-CO_2e)	0.66	1.02	1.68
15-yr Net change in C flux	(Tg C-CO_2e)	0.35	0.56	0.90

Supplementary Table 4. Lake area changes in the pan-Arctic during recent decades. Lake area changes including gross lake area loss (GLAL) and gross lake area gain (GLAG) show that Lake Change Flux Ratios (R_{LCF} = GLAL/GLAG) are below the threshold values of 4 and 7 (for non-yedoma and yedoma permafrost soil regions respectively; see Eq. 2 in Supplementary Note 2), suggesting that all net changes in lake area have likely led to a net increase in carbon emissions to the atmosphere during the historical satellite period. The exception is Smith et al. (93), in which coarse resolution imagery (80 m to 150 m) possibly led toward a bias in the detection of GLAL and underestimation of GLAG, and therefore likely not an accurate representation of R_{LCF}.

Reference	No.	Region	Study area (km^2)	Study period	Data source /resolution	Minimum lake size	GLAL (km^2)	GLAG (km^2)	Net lake area change (km^2)	Net lake area change (%)	R_{LCF} = GLAL/GLAG	Pre-drainage GLAL flux	Post-drainage GLAL + GLAG flux	Net flux change
												(unit mass C-CO_2e km^{-2} yr^{-1})		
Gross Lake Area Loss and Gain														
Nitze et al. 2017	24	Centra Yakutia	56,700	1999-2014	Landsat (30-m)		-48.28	501.2	452.9	+48.0%	0.10	-540	199,785	199,246
Boike et al. 2016	25	Lena River catchment in Yakutia, Russia	315,000	2002-2009	30-m TM/ETM	≥ 4 pixels	-28.62	976.0	947.4	+17.9%	0.03	-320	389,409	389,089
Sannel & Kuhry 2011	26	Hudson Bay Lowlands, Canada	4	1954-2006	0.6-1m IKONOS/QuickBird and aerial photos	1 pixel	-0.0770	0.1035	0.0265	+4.5%	0.74	-1	41	40
Sannel & Kuhry 2011	26	Rogovaya, NW Siberia	4	1974-2007	0.6-1m IKONOS/QuickBird and aerial photos	1 pixel	-0.2326	0.2339	0.0013	+0.2%	0.99	-3	92	90
Nitze et al. 2017	25	Kolyma Lowland, Siberia	73,339	1999-2014	Landsat (30-m)		-279.1	218.3	-60.83	-0.5%	1.28	-3,121	85,716	82,595
Carroll et al. 2011	27	Canada [50°N to 7[illegible]	-	2000-2009	250m	2-3 ha	-28,687	22,000	-6,687	-1.0%	1.30	-320,773	8,636,887	8,316,114
This study	-	N & NW Alaska (yedoma lakes)	82,954	1999-2014	Landsat (30-m)		-247.9	129.3	-118.7	-1.0%	1.92	-2,772	50,348	47,576
Jones et al. 2011	28	Northern Seward Penninsula, Alaska	700	1951-2000	1m, 1.7 m IKONOS and aerial photos	0.1 ha	-15.40	8.000	-7.400	-14.9%	1.93	-172	3,116	2,944
Nitze et al. 2017	24	Alaska North Slope	31,715	1999-2014	Landsat (30-m)		-77.86	39.37	-38.49	-0.7%	1.98	-871	15,321	14,451
Chen et al. 2014	29	Yukon Flats, Alaska (all lakes)	4,224	1984-2009	30-m TM/ETM	1 ha	-10.98	5.120	-5.860	-4.8%	2.14	-123	1,988	1,866
Nitze et al. 2017	24	AKS (Western Alaska)	31,135	1999-2014	Landsat (30-m)		-48.31	20.24	-28.07	-2.8%	2.39	-540	7,838	7,298
Hinkel et al. 2007	30	Plain, Barrow Penninsula,	3,600	1955-2002	5m aerial photos, ORRI radar	10 ha	-16.848	5.184	-11.66	-1.8%	3.25	-188	1,985	1,796
This study	-	N & NW Alaska (non-yedoma lakes)	429,451	1999-2014	Landsat (30-m)		-82.62	25.03	-57.59	-3.9%	3.30	-924	9,575	8,651
Sannel & Kuhry 2011	26	Tavvavouma, Sweden	1	1963-2003	0.5-1m IKONOS/QuickBird and aerial photos	1 pixel	-0.0347	0.0092	-0.0255	-19.4%	3.78	-0.4	3.5	3.1
Smith et al. 2005	31	Siberia	515,000	1970-2000	80-m MSS, 150-m Russian	40 ha	-930.0	130.0	-800.0	+12%; -12%†	7.15	-10,399	47,219	36,819

†12% increase in continuous permafrost zone; 12% decrease in discontinuous permafrost zone

Supplementary Table 4 cont.

Reference	No.	Region	Study area (km^2)	Study period	Data source /resolution	Minimum lake size	GLAL (km^2)	GLAG (km^2)	Net lake area change (km^2)	Net lake area change (%)	R_{LCF} = GLAL/GLAG
Net Lake Area Change Only											
Payette et al. 2004	32	Hudson Bay northeast coast, Quebec, Canada	0.1	1957-2003	aerial photos	1 pixel				+50%	n/a
Karlsson et al. 2012	33	7129 subbasin, Siberia	48,000	1973-2007/9	60 m, 30 m	10 ha				+25%	n/a
Christensen et al. 2004	34	Stordalen Mire, Abisko, Sweden	1	1970-2000	1 meter resolution aerial photos	1-m pixel				+4.9%	n/a
Walter et al. 2006	35	Cherskii, Siberia	12,000	1974-2000	Landsat MSS, 7 etm+	-				+14.7%	n/a
Karlsson et al. 2012	33	Nadym basin, Siberia	95,100	1973-2007/9	60 m, 30 m	10 ha				3%	n/a
Riordan et al. 2006	36	Arctic Coastal Plain, Alaska	260	1954-2000	30-m high-altitude aerial photos + satellite	0.2 ha				+1%	n/a
Plug et al. 2008	37	Tuktoyaktuk Penninsula, Canada	12,500	1978-2001	Landsat MSS, 7 ETM+	1.3 ha				~0	n/a
Edwards et al. 2016	38	Yukon Flats marginal upland, Alaska	Eight lakes	1978-2009	aerial photos + Ikonos satellite (1-m)	-				0%	n/a
Jones et al. 2009	39	National Petroleum Reserve Alaska	1,224,000	1985-2007	Landsat ETM, Landsat 7 ETM+	10 ha				-1%	n/a
Karlsson et al. 2012	33	Pur basin, Siberia	1,200	1973-2007/9	60 m, 30 m	10 ha				-2%	n/a
Labreque et al. 2009	40	Old Crow, Canada	5,600	1951-2000	15 m aerial photos and Landsat 7 ETM+	-				-3.5%	n/a
Riordan et al. 2006	36	Denali, Alaska	820	1954-2000	30-m high-altitude aerial photos + satellite	0.2 ha				-4%	n/a
Necsoiu et al. 2013	41	Kobuk Drainage, Alaska	140	1951-2005	~1m resolution aerial+satellite	0.09 ha				-5.9%	n/a
Veremeeva & Gubin 2009	42	Kolyma Lowland, Siberia	6,500	1973-2001	Landsat MSS, Landsat 7 ETM+	0.1 ha				-6%	n/a
Riordan et al. 2006	36	Yukon Flats, Alaska	2,020	1954-2000	30-m high-altitude aerial photos + satellite	0.2 ha				-18%	n/a
Riordan et al. 2006	36	Minto Flats, Alaska	810	1954-2000	30-m high-altitude aerial photos + satellite	0.2 ha				-25%	n/a
Roach et al. 2013	43	Alaska National Wildlife Refuges	-	1985-2007/9	30-m	0.58 ha		projected 50-year trend, -24.6% net change			
Rover et al. 2012	44	Central Alaska	15,000	1979-2009	60-m MSS, 30-m ETM+	0.1 ha		some increased, decreased, or stable			
Jepsen et al. 2013	45	Yukon Flats, Alaska	5,150	1979-2009	60-m MSS, 30-m ETM+	-		some increased, decreased, or stable			

Supplementary Table 5. Field-measured fluxes from thermokarst (TK) lakes and drained thermokarst lake basins (DTLB) in yedoma and non-yedoma permafrost regions of Alaska and Siberia. *n* is the number of lakes or DTLBs represented. Based on GWP_{100} of 28, we used a conversion factor, 10.2 to convert C-CH_4 fluxes to C-CO_2e fluxes (Supplementary Table 2).

			CH_4 Flux ($g\ C\ m^{-2}\ yr^{-1}$)				CO_2 Flux ($g\ C\ m^{-2}\ yr^{-1}$)				Flux (CH_4 + CO_2]
	Region	Data Sources	mean	s.e.m	(n)	median	mean	s.e.m	(n)	median	($kg\ C\text{-}CO_2e\ km^{-2}\ yr^{-1}$)
Yedoma permafrost soils											
Young TK lakes (<100 yrs)	Siberia, Alaska	23,46, This study	129.6	± 8.6	(13)	136.7	449.0 †			473.6 †	1,768,387
Mature TK lakes (>100 yrs)	Siberia, Alaska	23,35,46-48	9.5	± 3.3	(8)	8.8	180.8	± 72.2	(6)	121.0	277,164
DTLBs	Siberia, Alaska	6,35,49-50	4.8	± 2.6	(2)		-26.1	± 14.3	(8)		22,773
Non-yedoma permafrost soils											
Young TK lakes (<100 yrs)	Alaska	23,46	37.5	± 15.2	(5)	30.4	148.9 ‡			120.6 ‡	530,600
Mature TK lakes (>100 yrs)	Siberia, Alaska	23,46-47,51	6.9	± 6.5	(15)	0.4	53.5	± 4.5	(6)	54.7	124,159
DTLBs	Alaska	51	2.9	± 0.8	(36)		-29.5	± 25.0	(36)		-482
Terrestrial land ecosystems		52-54	1.0				-5.6				4,100

† Lacking field-measurements of CO_2 emissions specific to young yedoma thermokarst lakes, CO_2 values shown here are estimated from observed CH_4 emissions and based on the emission ratio [0.29 = g C-CH_4 m^2 yr^{-1} / C-CO_2 m^2 yr^{-1}] observed among five mature yedoma lakes in Alaska and Siberia where all modes of emission were measured, including ebullition, diffusion, ice-bubble storage and water-column storage.

‡Similar to †, CO_2 values shown for young non-yedoma thermokarst lakes are estimated from observed mature non-yedoma thermokarst lake CH_4 emissions and based on the observed emission ratio among mature six non-yedoma thermokarst lakes [0.25 = g C-CH_4 m^2 yr^{-1} / C-CO_2 m^2 yr^{-1}].

Supplementary Table 6. CH_4 and CO_2 flux ratios observed in field work between lake change land units. Ratios were calculated from flux values in Supplementary Table 5. Land units include pre-drainage open water lake surfaces (Mature TK), post-drainage thermokarst lake basins (DTLBs), thermokarst-expansion zones (TK lakes), and land. For drainage, positive values indicate that a landscape shift from open water to a drained lake basin represents emissions to the atmosphere, while negative fluxes indicate uptake of atmospheric carbon.

Permafrost soil type	Drainage (DTLB flux / Mature TK lake flux)	Expansion (Young TK lake flux / Land flux)
Yedoma	+0.082	+430
Non-yedoma	-0.004	+129

Supplementary Notes

Supplementary Note 1. Carbon fluxes associated with plant colonization in drained lake basins. Our AThaw calculations include drainage of lakes formed since 1950, with the assumption that once drained, the lakes no longer emit CH_4 or CO_2. Our calculations do not include fluxes associated with new vegetation colonization in drained lake basins, which has the potential to change the carbon flux in that part of the landscape. Here we consider the potential implications of new vegetation colonization in drained lake basins on our AThaw model results. Previous modeling and empirical studies have shown that the magnitude and sign of the fluxes in drained lake basins depend on time since drainage because vegetation succession follows changes in soil conditions. On the Alaska North Slope, young (0 to 50 years old) vegetated drained basins are first colonized by wet graminoid (*Arctophila fulva*) and sedge (*Dupontia fisheri* and *Eriophorum scheuchzeri*) vegetation[55-56], productive plant types associated with the highest CH_4 fluxes among arctic peatlands[57]. Over time during the Holocene, freezing and cracking of the drained lake sediments as permafrost aggraded resulted in the formation of ice-wedge polygons, among which elevated rims in older basins (300 to 2000 years) are covered by *Dicranum elongatum* and depressed wet centers support *A. fulva*, *Carex aquatilis* and *Sphagnum spp*. We have observed similar patterns of vegetation succession in the Yedoma thermokarst-lake landscapes of Siberia and Alaska, with *Calamagrostis canadensis*, *D. fisherii*, and *C. aquatilis* establishing in wet, drained lake basins less than 50 years old. Permafrost aggradation over time leads to lower gross primary productivity of plants colonizing ice-wedge polygon rims (*C. bigelowii*, *Eriophorum angustifolium* and *Sphagnum fuscum*, *Betula nana*, *Salix* sp., and prostrate ericaceous shrubs) with low-centered polygonal ponds in older basins (2,000 to >5,000 years) containing productive species such as *C. aquatilis*[58]. Long-term frost heave, including pingo formation, can result in drying of basin centers; and since topographic relief is higher in yedoma landscapes, those basins with a steep topographic gradient also become drier.

Modeling, paleoenvironmental analysis, and eddy covariance tower studies have shown that millennial-scale thermokarst-lake landscape evolution results in long-term (many centuries to thousands of years) net carbon sinks in some drained lake basins of the Yedoma region[49,59], and net carbon sources in others, such as drained lake basins on the Alaska North Slope[51]. A much shorter time frame following lake drainage is relevant to our AThaw study period (~150 years, i.e. 1950 to 2100). Landscape-scale summer flux measurements in young (<300 years) drained lake basins show that the CO_2e flux when both CO_2 and CH_4 fluxes are accounted for, hovers between neutral and a positive net source to the atmosphere, despite significant uptake of atmospheric carbon by newly established vegetation[49,55,60]. As in other northern high-latitude landscapes[61], accounting for winter fluxes in drained lake basins can cause a sign change from net uptake to net annual emissions in recently drained lake basins[60]; hence we assume that all drained basins <300 years in our study are a net annual source of atmospheric carbon. While permafrost may aggrade in many drained lake basins that form following drainage this century under RCP4.5 such that fluxes in present-day basins may be relevant in a RCP4.5 future, the more extreme warming predicted for RCP8.5 will inhibit re-freezing of taliks[62], allowing the potential for additional CH_4 emissions from these taliks. Incorporation of such drained lake basin vegetation and talik carbon fluxes in AThaw would further strengthen our conclusions that the magnitude of the 21st-century permafrost carbon feedback is larger when the processes of abrupt thaw beneath lakes and their subsequent drainage are accounted for.

Supplementary Note 2. Regional lake water balance impact on carbon budget. Remote-sensing studies report a diversity of lake-change trends in the Arctic during recent decades, attributable to differences in regional climate, permafrost, glaciation history, surface geology, and overall landscape characteristics. In some regions, large (e.g. +48%) net increases in lake area are observed. In many regions, there is a small net decrease in lake area (Supplementary Table 4). However, resolution of remote-sensing base images also impacts results since the loss of lake area affecting numerous contiguous pixels is readily detected in course-resolution imagery (e.g. 30-m to 120-m resolution), while formation of small, new lakes can only be reliably detected with fine resolution (e.g. 1-m) imagery.

The formation of new thermokarst lakes during recent decades often goes unnoticed in Landsat analyses covering a short time period at ≥30m resolution (Supplementary Fig. 3). This process of new lake formation and growth in permafrost uplands is likely to accelerate in a warmer climate[62-64]. The important question is whether current upland geomorphology can sustain lateral growth of new lakes to persist through the 21st century on valley-fragmented uplands. Based on average expansion rates observed with high resolution remote sensing data during the past 60 years (0.3 m yr^{-1}) and proximity of newly formed lakes to drainage channels[28], most of these newly formed lakes are likely to survive this century. In a much warmer Arctic (RCP4.5 and RCP8.5), not just the flat uplands will thaw, but also the older drained lake basins containing lots of ground ice now protected by a layer of peat. Hence, in a much warmer and moister Arctic, we will likely see new lakes forming in older drained lake basins and remnant lakes rapidly expanding.

How likely these lakes are to drain depends on local factors such as basin morphology, ice content, permafrost thickness, and proximity to drainage channels. Nitze et al. (24) showed that a slight net lake area decrease in many regions of the Arctic during 1999

to 2014 was predominately driven by the drainages of a small number of very large single lakes, where single events have a very big impact on the overall lake area balance. However, those lakes are not likely to be large CH_4 emitters today since they will have depleted their supply of labile carbon in thawed permafrost earlier in their development several thousand years ago[17]. It is the talik formation beneath new small lakes as well as talik expansion of existing lakes thawing and mobilizing old permafrost sediments that fuels CH_4-producing microbes. This process of gross lake area growth occurs in the majority of lakes on the landscape[28,65-66] and strongly counterbalances gross lake area losses (Supplementary Table 4).

Supplementary Table 4 summarizes the literature on historic and present-day lake area change dynamics and associated carbon flux implications. Calculating the carbon response to lake area change is not possible from net lake area change alone; it requires knowledge of gross lake area loss (GLAL) and gross lake area gain (GLAG). We applied field-work observed carbon flux factors associated with lake drainage (i.e. GLAL) and lake formation and expansion (i.e. GLAG) (Supplementary Table 5) to historically observed lake area changes (Supplementary Table 4). This showed that despite regional net lake area loss, a region will still exhibit a net increase in carbon emissions associated with lake change since carbon fluxes on the drained portion of the landscape are small in comparison to fluxes associated with lake-area expansion that taps into the soil carbon pool across a thick column of thawing permafrost (Supplementary Table 6).

For gross lake area expansion fluxes to offset gross lake area loss fluxes, their area-weighted CO_2e fluxes must be equal, i.e.,

$$\Delta F_{GLAG} \times A_{GLAG} = \Delta F_{GLAL} \times A_{GLAL} \qquad (1)$$

where ΔF_{GLAG} is the change in flux (g C-CO_2e per unit area per unit time) in those areas of the landscape (A_{GLAG}) where lake expansion (GLAG) occurs, and ΔF_{GLAL} is the change in flux (g C-CO_2e per unit area per unit time) in those areas of the landscape (A_{GLAL}) where lake drainage (GLAL) occurs. For this to hold the *Lake Change Flux Ratio*, R_{LCF}

$$R_{LCF} = \frac{\Delta F_{GLAG}}{\Delta F_{GLAL}} = \frac{A_{GLAL}}{A_{GLAG}} \qquad (2)$$

Applying observed landscape unit fluxes in Supplementary Table 5 to Equation 1 shows that until gross lake drainage area is approximately four and seven times gross lake expansion area (*Lake Change Flux Ratio*, R_{LCF} = 4.2 and 6.9 for non-yedoma and yedoma permafrost soil types, respectively), a ratio yet to be observed studies of historical lake change (Supplementary Table 4), the relatively high emissions associated with mobilization of thawed permafrost carbon in thermokarst expansion zones (GLAG) will lead to a net increase in regional atmospheric carbon emissions associated with lake change.

Supplementary Discussion

Abrupt thaw hotspots of CH_4 emission

Once formed, thermokarst lakes strongly alter the local thermal balance by transferring heat from the water body to the underlying ground more effectively than other land cover types[67]. When thermokarst lakes grow deep enough to prevent lake-ice grounding and thus winter-refreeze of underlying sediments, thaw rates of permafrost below lakes speed up dramatically as above-zero temperatures prevail year-round at the lake bottom. This rapid, downward permafrost thaw and talik formation beneath the lake[68] (Fig. 1a) creates an anaerobic environment where permafrost soil carbon can be rapidly mineralized by microbial communities and released as CH_4 and CO_2 (17). Methane produced in dense, thawed sediments escapes lakes primarily by ebullition (bubbling) seeps[35,69]. Radiocarbon dating combined with year-round flux measurements suggest that particularly strong thermokarst-lake ebullition seeps, termed hotspots[35], originate from the greatest depths in closed-system taliks, along the downward and laterally expanding thaw front[70].

Our mapping of CH_4 hotspot seep locations in Goldstream Valley lakes showed no large hotspot ebullition seeps occurring in lakes that have not significantly expanded since 1949 (Supplementary Fig. 8a-d); hotspot seeps predominately occurred in areas of lakes that experienced abrupt thaw by thermokarst shore expansion since 1949 (Supplementary Fig. 8e, f); and large seeps were distributed across whole lake surfaces in lakes entirely new since 1949 (Supplementary Fig. 8g-j). Radiocarbon ages of CH_4 emitted by large ebullition seeps are directly related to the age of deep, organic rich permafrost soils surrounding the lakes[23], suggesting that decomposition of deeply thawed permafrost carbon beneath lakes fuels CH_4 production in the abrupt thaw environment. The spatial pattern of exceedingly high CH_4 emissions in lake zones recently formed by active thermokarst and diminishing emissions toward

zones of lakes that have remained stable open water with presumably little vertical thaw during the past 60-years, or where carbon-rich sediment layers were previously thawed through[71], supports AThaw model assumptions that the labile fraction of the permafrost carbon pool is mineralized beneath lakes over time scales of years to decades.

Supplementary Methods

Radiative forcing

The annual global instantaneous radiative forcing due to total net permafrost-region surface CO_2 and CH_4 fluxes is directly proportional to the perturbation concentrations in the atmosphere (e.g. 72). We simulated the instantaneous radiative forcing impact on the atmosphere by using impulse-response functions[73] to represent the net soil and thermokarst-lake CO_2 and CH_4 fluxes as a perturbation to an otherwise constant atmosphere. A constant or time-varying net CO_2 flux (emission or uptake), $\Phi_{CO2}(t)$, since an arbitrary start time, t = 0, causes a perturbation to the atmospheric CO_2 burden at any time t that is given by

$$CO_2(t) = \sum_{i=1}^{4}\left(f_i \cdot \int_0^t \Phi_{CO2}(t')\, e^{(t'-t)/\tau_i} dt'\right) \quad (3)$$

where f_i is the fraction of the flux added to (if net emission) or removed from (if net uptake) CO_2 reservoir i, which has a lifetime of τ_i. The parameterization of Eq. 3 is developed by fitting the model response to the carbon cycle behavior of Earth System Models[73]. We use a modification of the mean model parameter values reported by Joos et al. (73), which they based on fitting this model to the carbon cycle dynamics of 15 earth system models including full Earth System Models, Earth System Models of Intermediate Complexity (EMICs), and box-type models. We set the lifetime of the slowest turnover time [τ_4 = infinity in Joos et al. (73)] to 200,000 years, related to a re-equilibration of the weathering-burial component of the carbon cycle[74-75]. This modification has little impact on the model behavior over 100-year simulations. For CH_4, the atmospheric perturbation due to net flux $\Phi_{CH4}(t)$ is given by

$$CH_4(t) = \int_0^t \Phi_{CH4}(t') e^{(t'-t)/\tau_{CH4}}\, dt' \quad (4)$$

For CH_4 we use a lifetime, τ_{CH4}, of 12.4 years[76].

For CH_4, radiative forcing is a product of three terms: CH_4 atmospheric perturbation (Eq. 4), methane's radiative efficiency (3.63 x 10^{-4} W m^{-2} ppb^{-1} CH_4) and an indirect effects multiplier of 1.65 ± 0.3 (76). For CO_2, radiative forcing is the product of its atmospheric perturbation (Eq. 3) and its radiative efficiency (0.137 x 10^{-4} W m^{-2} ppb^{-1} CO_2). We convert between ppb and mass using 2.78 Tg CH_4 per ppb (77), and, by molar equivalence, 7.65 Tg CO_2 per ppb. We evaluated the instantaneous radiative forcing calculations by driving the impulse-response model with 100 kg pulse emissions of CO_2 and CH_4, and then computing the resulting 20-year and 100-year global warming potential (GWP) values by integrating (summing) the resulting CO_2 and CH_4 radiative forcings over 20 or 100 years, and computing the CH_4:CO_2 ratio (i.e., methane's GWP). This model parameterization generated GWP_{20} = 85 kg CO_2e/kg CH_4, and GWP_{100} = 28 kg CO_2e/kg CH_4, comparable to IPCC AR5 reported values that exclude carbon-cycle feedbacks [GWP_{20} = 84 and GWP_{100} = 28; Table 8.7 in Myhre et al. (76)].

Optical remote sensing mapping of new thermokarst lakes

To quantify the 2-D spatial extent of abrupt thermokarst-lake formation since 1949 and to quantify areas of lake expansion where rapid talik formation takes place, we mapped historic lake margins in Goldstream Valley, Alaska using high-resolution (1 m spatial resolution) single-band airborne optical images from 1949, 1967, and 1985 and more recent lake margins using high-resolution (2.5 m) orthomosaiked multi-band 2010 SPOT satellite images. SPOT imagery consisted of visible (blue, green and red) and near-infrared bands. We transformed the four SPOT bands using Principal Component Analysis (PCA) into linearly uncorrelated PC bands that are orthogonal to each other. This removed any redundant spectral information from all four bands and helped to enhance images by separating water bodies more distinctly from the surrounding landscape features[78].

To map lake margins, we applied Object-based Image Analysis (OBIA) that first segments the image into meaningful groups of pixels based on their spatial and spectral homogeneity called image objects and then classifies image objects[79-80]. One of the advantages of OBIA is that it allows classification of image objects based on spectral, spatial, textural, shape and contextual information pertaining to them, which can improve image classification for spatially contiguous landscape features such as lakes, compared to traditional pixel-based classification techniques.

For historic lake margin delineation, we segmented the airborne image into image objects followed by simple threshold classification. We chose a threshold value on mean brightness or spectral value of the image object to map water bodies. For recent lake margin delineation from SPOT imagery, we applied segmentation on visible, near-infrared and the first two PC bands that carried

the most variance (>98%). We used thresholds on the first PC band and the Normalized Difference Water Index (NDWI) (81), an index based on the green and near-infrared band, to map water bodies. Thresholds varied from scene to scene due to variation in water body types and radiometric properties of the images. Finally, we manually removed non-lake water bodies (rivers, streams), terrain shadows, and other similar misclassified image objects.

Airborne electromagnetic imaging of abrupt thaw

To detect taliks underneath lakes that formed within recent decades (Figs. 1a and 5, Supplementary Fig. 7) and to estimate talik depths, we determined permafrost geometry using airborne electromagnetic (AEM) data collected and analyzed following methods used previously in Alaska[82-85]. In March 2016 approximately 2,000 line km of CGG RESOLVE frequency domain AEM data were collected over the Goldstream Creek watershed in interior Alaska[86]. This AEM system records six frequencies of inphase and quadrature data using six pairs of wire loops. Coplanar data were recorded at 900 Hz, 1800 Hz, 8200 Hz, 40 KHz, and 140 KHz; coaxial data were recorded at 3300 Hz. The system was housed in a 9 m bird and was towed on a 30 m long line from an Eurocopter A350B3 helicopter.

From the preliminary data, 1D resistivity models without lateral spatial constraint were created using University of British Columbia's EM1DFM inversion software[87] (Supplementary Fig. 7). After removing cultural noise, spatially constrained 1D resistivity models were created from the electromagnetic data using Aarhus' Workbench inversion software[88]. These 1D models were gridded with Paradigm's GOCAD software[89] to create a 3D resistivity model (Fig. 5e). The combination of spatially constrained inversions and 3D gridding create a laterally consistent 3D model from 1D inversions at the expense of resolution. In this case the talik beneath lake II is less visible in the extracted cross section from the 3D model presented in Fig. 5e compared to the unconstrained EM1DFM 1D models along at the actual measurement locations (flight lines) in Supplementary Fig. 7. Along-line down sampling of the data to 30 meters from 3 meters further decreases the lateral resolution in the upper most potion of both the Workbench and EM1DFM generated models.

Apparent differences in boundary interpretation of thawed and degraded permafrost between Lake I and Lake II in Fig. 5e are an artifact of modeling. In the modeling process, the thicker the upper conductive area (thawed area) the more difficult it is to see a resistive (frozen area) area below it. If a resistive area exists it may show higher resistivity in the modeling process than an area with similar physical properties and no conductive layer above it. There is an increase in the resistivity in the model with depth under Lake I suggesting a decrease in free water and temperature. Another way to view the interpretation is Lake I has a thermal footprint laterally and below the actual thawed area and a much larger footprint than Lake II; the first 30 meter subsurface below Lake I is different than the subsurface 30 to 100 meters in depth. The model also supports that the frozen material is likely colder and thicker below lake II than lake I.

Information about the subsurface was derived from resistivity models based on the published resistivity ranges of materials[90-93]. Data from more than 60 boreholes distinguishing frozen from unfrozen material within the immediate study area[94-95] were also used. Initial interpretation indicates cold, stable permafrost has high resistivity (>500 Ohm-m); degrading permafrost can have moderate resistivity (200 – 500 Ohm-m); and thawed areas and lakes have low resistivity (< 200 Ohm-m). These values are subject to change with further analysis of subsurface data in the study area and are dependent on frozen material type (e.g. silt, gravel)[90-93]. The depth of exploration for these AEM data is 50 to 150 m, dependent on subsurface resistivity. Lateral resolution is approximately 30 m; vertical resolution is a few meters at the surface to tens of meters at the bottom of the models.

Remote sensing large-scale Alaska lake water balance from 1999-2014

We quantified net lake area changes, gross lake area gain (GLAG), and gross lake area loss (GLAL) across an extensive (~512,000 km^2) permafrost region of Alaska to identify areas of potential rapid talik formation. We mapped thermokarst-lake changes with a hybrid approach of Landsat trend calculation, machine-learning classification and object-based image analysis based on Nitze et al. (24). First, we calculated robust trends of several multi-spectral indices (Tasseled Cap, NDVI, NDMI, NDWI) of Landsat peak summer season data from 1999-2014 based on the methodology of Nitze & Grosse (96). To classify the spectral-temporal trend signatures into semantic lake change information, we applied a Random Forest machine-learning algorithm[97]. The classification model was trained with trend information at extensive ground truth locations across several biogeographical regions within the northern hemisphere permafrost zone[24]. Based on the classification results, we then extracted individual lakes larger than one hectare with zones of stable water as well as zones of lake area gain or loss. Therefore, the minimum mapping unit is 1 ha. To calculate lake area changes we used classification probabilities within the dynamic lake zones to account for subpixel changes. Gridded results were calculated for the spatial representation of gross lake growth. Each region was subdivided into 7.5 km x 7.5 km large squares and

GLAG, GLAL and net lake change per pixel within each grid cell were summed. Finally, the percentage of lake area change (land-lake transition) per grid cell was calculated as a fraction per grid cell.

We calculated carbon flux changes by multiplying field-observed flux factors (Supplementary Table 5) to gross lake expansion and gross lake drainage rates (Supplementary Table 3). To account for the carbon fluxes of yedoma and non-yedoma permafrost soils, we grouped lakes into yedoma and non-yedoma lakes based on the spatial intersection of individual lake centroids with the Database of Ice-Rich Yedoma Permafrost dataset[98]. Our net carbon emission calculation assumes linear expansion and drainage over the observation period. This assumption is likely more valid for expansion than drainage since expansion is an ongoing process that affects a large number of lakes in the dataset, while drainage is more stochastic and tends to affect only a small number of individual lakes[28]. Given that changes in carbon fluxes associated with drainage are orders of magnitude smaller than those associated with expansion (Supplementary Table 5), applying non-linear drainage rates would not have a large impact on our results.

Field measurements of volumetric ebullition flux

Methane, a semisoluble gas, readily forms bubbles in lake sediments, while the more soluble CO_2 tends to remain in solution and escape arctic lakes by diffusion[46]. Newly-formed CH_4-rich bubbles often follow existing escape pathways through sediments[99], resulting in point-sources of continuous ebullition seepage at the sediment-water interface. During the ice-free season, bubbles rise through the water column and escape to the atmosphere. In winter, surface lake ice seasonally traps seep bubbles, although the majority of winter-bubble methane escapes lakes in spring as ice melts[100]. Using field work, we estimated ebullition from lake sediments associated with discrete seeps (Fig. 1b) following the lake-ice ebullition survey method of Walter Anthony *et al.* (47). We estimated lakes CO_2 emissions in Fig. 1b. based on the ratio of CH_4 to CO_2 emissions measured in 40-lakes along a north-south Alaska transect[46].

Ebullition seeps were identified in field work as A, B, C, and Hotspot types according to patterns of bubbles trapped in early winter lake ice. The lake ebullition seep-classification system has been described in detail in previous publications[35,47,70,101]. Briefly, A-type ebullition seeps are relatively small clusters of ebullition bubbles in which individual bubbles stack on top of each other in the winter ice sheet without merging laterally. Due to progressively higher ebullition rates, individual bubbles of B-type seeps laterally merge into larger bubbles under the ice prior to freezing in ice. Types A and B seeps produce low gas-volume clusters of bubbles in lake ice with cluster diameters typically <40 cm. The larger C seeps result in large (usually >40 cm diameter) gas pockets in ice separated vertically by ice layers containing few or no bubbles. Bubble-trap measurements showed that the solid ice layers in between the large gas pockets of C-type seeps represent periods of relative quiescence in between large ebullition events[70,101]. Hotspot seeps have the greatest mean daily bubbling rates. The frequency of ebullition release from hotspot seeps and the associated water column convection created by rising bubble plumes maintains mostly ice-free holes in winter lake[100,102].

We removed snow from early winter lake ice to expose ebullition bubble clusters trapped in ice for seep classification. We quantified seep density, GPS-mapped seeps, and measured fluxes and collected gas using submerged bubble traps. On foot, we surveyed 4,914 individual seeps within 65 plots (30 to 640 m^2 per plot, average of five plots per lake). Ice-bubble survey plots were typically 1-m wide transects originating from margins, oriented perpendicular to the shores and extending toward the lake center. Additional lake-center transects were also surveyed. We calculated seep density within each lake as the total number of seeps surveyed within each lake divided by area surveyed.

To convert seep densities to estimates of year-round volumetric ebullition we measured ebullition fluxes year-round using submerged bubble traps. In some lakes within each region, ice was opened above the seeps for placement of submerged bubble traps over the seeps in winter. Semi-automated bubble traps remained in place over individual seeps year round[101], providing daily and seasonal bubbling rates for individual seeps. Fluxes from other seeps were measured over the short term (<6 days) using manual bubble traps. Seep fluxes did not vary by type among regions[47]. Altogether the seep flux data set consists of ~210,000 individual flux measurements made using submerged bubble traps placed over ebullition seeps year-round. Seep class-specific flux rates measured on a subset of seeps were applied to all mapped seeps to estimate regional seep ebullition rates. In a recent comparison of methods, Walter Anthony & Anthony (47) showed that when at least three 50-m transects per lake are used to quantify seep ebullition, the estimate of mean whole-lake ebullition is 4-5 times more accurate than the mean flux determined by placement of ~20 0.2-m^2 bubble traps randomly distributed across lake surfaces.

Whole-lake mapping of CH_4 hotspots

To assess the distribution of CH_4 hotspot-type seeps across entire lakes and within their abrupt thaw thermokarst zones, we mapped large CH_4 seeps manifested as ice-free holes in early winter and late spring lake ice across the entire surfaces of Goldstream Valley lakes using two approaches. First, we conducted ground surveys following a ~10-m wide grid pattern in lake ice to map with

DGPS the locations of ice-free holes formed by high rates of CH_4 ebullition. In addition to ground surveys, on a subset of lakes we also mapped ice-free holes in lake ice (i.e. hotspot seeps) which appears as black circular features on white, snow-covered winter lake aerial photos[71]. We used fine-scale (10 cm spatial resolution) optical airborne images, which consisted of three visible bands (blue, green and red). We applied PCA on all three visible bands for image enhancement to increase the contrast between the dark open-holes and the background snow-covered lake ice. Similar to SPOT image water body mapping, for open-hole CH_4 seep mapping, we segmented images using the visible bands and the first two PC bands that carried the most variance (>98%). Then we applied threshold values to the first PC band to map open-hole hotspots followed by manual removal of misclassified objects.

Bubble CH_4 concentration and radiocarbon dating

To determine mass-based estimates of CH_4 ebullition, we applied region-specific measurements of bubble CH_4 concentrations to the individual lakes where seep-bubble gases were collected and measured following methods detailed by Walter Anthony *et al.* (103) and Greene *et al*. (100). Briefly, gases were either collected directly from pockets of gas trapped in lake ice or from fresh ebullition events using submerged bubble traps placed near the surface of the water column above seeps. Bubble gas was collected into 20-ml or 60-ml glass serum vials, sealed with butyl rubber stoppers, and stored under refrigeration in the dark until laboratory analyses. We measured bubble CH_4 and CO_2 concentrations using a using a GC-2014 gas chromatograph (Shimadzu, Addison, Illinois, USA) equipped with a flame ionization detector and a PLOT alumina column (detector temperature 250 °C, oven 40 °C, high-purity helium as carrier gas) at the University of Alaska Fairbanks. Up to 246 individual seep ebullition events were measured per lake. In lakes where few or no seep seep-bubble gas concentrations were determined, we applied mean values of methane concentration by seep class from all study lakes[47]: A, 73% CH_4; B, 75% CH_4; C, 76% CH_4; Hotspot 78% CH_4. Carbon dioxide concentration in seep ebullition bubbles was 0.61 ± 0.45% by volume (mean ± standard deviation).

We determined the radiocarbon age of methane in lake bubbles collected from seeps. In total 72 ebullition events from 11 thermokarst lakes in different regions of Alaska and Siberia were dated (Fig. 1c). Subsamples of bubbles were purified and combusted on a helium gas stream as described in Chanton *et al.* (104). Carbon dioxide in the samples was removed in a pre-combustion trap placed in liquid nitrogen. Methane was then combusted to CO_2 by passing it over copper oxide at 800 °C. Prior, the copper oxide was charged with oxygen at 600°C overnight. Following combustion, water was removed in pentane-liquid nitrogen slush, and the CO_2 was then trapped in a liquid nitrogen trap. Carbon dioxide was then purified by running it though another water trap; its volume was measured in a pressure manometer; and it was then sealed in a 6 mm Pyrex tube. Samples were then catalytically reduced to graphite[105], and the $^{14}C/^{12}C$ isotopic ratios were measured by accelerator mass spectrometry at the Woods Hole Oceanographic Institution's National Ocean Sciences AMS (NOSAMS) Facility. Additional samples were analyzed at the Keck Carbon Cycle AMS Facility at the University of California, Irvine. Calibration samples of ^{14}C dead tank CH_4 were prepared and analyzed as were samples yielding a result of 0.004 ± 0.0009 fraction modern (n=8).

Uncertainties associated with our field-based ebullition estimates were calculated by propagating spatial errors associated with ice-bubble surveys, which are a function of survey area and ebullition seep density[47], spatiotemporal errors associated seep class-specific bubble-trap flux measurements, and errors associated with bubble CH_4 concentrations.

Supplementary References

1. Koven, C. D., Lawrence, D. M., & Riley, W. J. Permafrost carbon-climate feedback is sensitive to deep soil carbon decomposability but not deep soil nitrogen dynamics. *PNAS* **112**, 3752-3757 (2015).
2. Koven, C. D. et al. A simplified, data-constrained approach to estimate the permafrost carbon–climate feedback. *Phil. Trans. R. Soc. A*. **373**, DOI: 10.1098/rsta.2014.0423 (2015).
3. Hugelius G et al. Estimated stocks of circumpolar permafrost carbon with quantified uncertainty ranges and identified data gaps. *Biogeosci*. **11**, 6573–6593 (2014).
4. Schneider von Deimling, T. et al. Observation-based modelling of permafrost carbon fluxes with accounting for deep carbon deposits and thermokarst activity. *Biogeosci*. **12**, 3469-3488 (2015).
5. Strauss, J. et al. The deep permafrost carbon pool of the Yedoma region in Siberia and Alaska. *Geophys. Res. Lett*. **40**, 6165–6170 (2013).
6. Walter Anthony, K. M. et al. A shift of thermokarst lakes from carbon sources to sinks during the Holocene epoch. *Nature* **511**, 452–456 (2014).
7. Dutta, K., Schuur, E. A. G., Neff, J. C., & Zimov, S. A. Potential carbon release from permafrost soils of Northeastern Siberia. *Glob. Change Biol*. **12**, 2336–2351 (2006).

8. Burke, E. J., Hartley, I. P., & Jones, C. D. Uncertainties in the global temperature change caused by carbon release from permafrost thawing. *The Cryosph*. **6**, 1063–1076 (2012).
9. Schädel, C. et al. Circumpolar assessment of permafrost C quality and its vulnerability over time using long-term incubation data. *Glob. Change Biol*. **20**, 641–652 (2014).
10. Sitch, S. et al. Evaluation of ecosystem dynamics, plant geography and terrestrial carbon cycling in the LPJ dynamic global vegetation model. *Glob. Change Biol*. **9**, 161–185 (2003).
11. Koven, C. D. et al. Permafrost carbon-climate feedbacks accelerate global warming. *Proc. Natl. Acad. Sci*. **108**, 14769–14774 (2011).
12. Lee, H., Schuur, E. A. G., Inglett, K. S., Lavoie, M., & Chanton, J. P. The rate of permafrost carbon release under aerobic and anaerobic conditions and its potential effects on climate. *Glob. Change Biol*. **18**, 515–527 (2012).
13. Walter, B. P. & Heimann, M. A process-based, climate-sensitive model to derive methane emissions from natural wetlands: Application to five wetland sites, sensitivity to model parameters, and climate. *Glob. Biogeochem. Cyc*. **14**, 745–765 (2000).
14. Frauenfeld, O. W., Zhang, T., Barry, R. G., & Gilichinsky, D. Interdecadal changes in seasonal freeze and thaw depths in Russia. *J. Geophys. Res.-Atmos*. **109**, D05101, doi:10.1029/2003JD004245 (2004).
15. Hayes, D. J. et al. The impacts of recent permafrost thaw on land–atmosphere greenhouse gas exchange. *Environ. Res. Lett.* **9**, 045005, doi:10.1088/1748-9326/9/4/045005 (2014).
16. Schaphoff, S. et al. Contribution of permafrost soils to the global carbon budget. *Environ. Res. Lett*. **8**, 014026, doi:10.1088/1748-9326/9/8/085003 (2013).
17. Kessler, M. A., Plug, L., & Walter Anthony, K. Simulating the decadal to millennial scale dynamics of morphology and sequestered carbon mobilization of two thermokarst lakes in N.W. Alaska. ***J. Geophys. Res. Biogeosci.*** **117**, doi:10.1029/2011JG001796 (2012).
18. Kaufman, D. et al. Holocene thermal maximum in the western Arctic (0-180oW). *Quat. Sci. Rev*. **23,** 529-560 (2004).
19. Velichko, A. A. et al. Climate changes in East Europe and Siberia at the Late glacial–holocene transition. *Quat. Int*. **91**, 75–99 (2002).
20. Marcott, S. A., Shakun, J. D., Clark, P. U., & Mix, A. C. A Reconstruction of Regional and Global Temperature for the Past 11,300 Years. *Science* **339**, 1198–1201 (2013).
21. Walter, K. M., Edwards, M. E., Grosse, G., Zimov, S. A., & Chapin, F. S. Thermokarst lakes as a source of atmospheric methane during the last deglaciation. *Science* **318**, 633-636 (2007).
22. Brosius, L. S. et al. Using the deuterium isotope composition of permafrost meltwater to constrain thermokarst lake contributions to atmospheric methane during the last deglaciation. *J. Geophys. Res. Biogeosci*. **117**, G01022, doi:10.1029/2011JG001810 (2012).
23. Walter Anthony, K. M. et al. Methane emission proportional to permafrost carbon thawed in Arctic lakes since the 1950s. *Nat. Geosci*. **9**, 679-682 (2016).
24. Nitze, I. et al. Landsat-based trend analysis of lake dynamics across northern permafrost regions. *Rem. Sens*. doi.org/10.3390/rs9070640 (2017).
25. Boike, J. et al. Satellite-derived changes in the permafrost landscape of central Yakutia, 2000-2011: Wetting, drying, and fires. *Glob. Planet. Change* **139**, 116-127 (2016).
26. Sannel, A .B. K. & Kuhry, P. Warming-induced destabilization of peat plateau/thermokarst lake complexes. *J. Geophys. Res*. **116**, doi.org/10.1029/2010JG001635 (2011).
27. Carroll, M. L., Townshend, J. R. G., DiMiceli, C. M., Loboda, T., & Sohlberg, R. A. Shrinking lakes of the Arctic: Spatial relationships and trajectory of change. *Geophys. Res. Lett*. **38**, L20406 (2011).
28. Jones, B. M. et al. Modern thermokarst lake dynamics in the continuous permafrost zone, northern Seward Peninsula, Alaska. *J. Geophys. Res. Biogeosci*. **116,** G00M03 (2011).
29. Chen, M., Rowland, J. C., Wilson, C. J., Altmann, G. L. & Brumby, S. P. Temporal and spatial pattern of thermokarst lake area changes at Yukon Flats, Alaska. *Hydrol. Process*. **28**, 837–852. (2014)
30. Hinkel, K. M. *et al.* Methods to assess natural and anthropogenic thaw lake drainage on the western Arctic coastal plain of northern Alaska. *J. Geophys. Res*. **112**, F02S16 (2007).
31. Smith, L. C., Sheng, Y., MacDonald, G. M., & Hinzman, L. D.: Disappearing arctic lakes. *Science* **308**, 1429–1429 (2005).
32. Payette, S, Delwaide, A., Caccianiga, M., & Beauchemin, M. Accelerated thawing of subarctic peatland permafrost over the last 50 years. *Geophys. Res. Lett*. **31**, L18208, (2004).

33. Karlsson, J. M., Lyon, S. W., & Destouni, G. Thermokarst lake, hydrological flow and water balance indicators of permafrost change in Western Siberia. *J. Hydrol*. **464–465**, 459–466 (2012).
34. Christensen, T. R., Johansson, T., Akerman, H. J., & Mastepanov, M. Thawing sub-arctic permafrost: Effects on vegetation and methane emissions. *Geophys. Res. Lett*. **31**, L04501, (2004).
35. Walter, K. M., Zimov, S. A., Chanton, J. P., Verbyla, D., & Chapin, F. S. Methane bubbling from Siberian thaw lakes as a positive feedback to climate warming. *Nature* **443,** 71-75 (2006).
36. Riordan, B., Verbyla, D., & McGuire, D. Shrinking ponds in subarctic Alaska based on 1950–2002 remotely sensed images. *J. Geophys. Res*. **111**, doi.org/10.1029/2005JG000150 (2006).
37. Plug, L. J., Walls, C. & Scott, B. M. Tundra lake changes from 1978 to 2001 on the Tuktoyaktuk Peninsula, western Canadian Arctic. *Geophys. Res. Lett*. **35**, L03502 (2008).
38. Edwards, M., Grosse, G., Jones, B., & MacDowell, P. Deep thermokarst lakes in the Yukon Flats, Alaska. *Sediment. Geol*. in press doi.org/10.1016/j.sedgeo.2016.01.018 (2016).
39. Jones, B. M. *et al.* Arctic Lake Physical Processes and Regimes with Implications for Winter Water Availability and Management in the National Petroleum Reserve Alaska. *Environ. Management* **43**,1071–1084 (2009).
40. Labrecque, S., Lacelle, D., Duguay, C. R., Lauriol, B., & Hawkings, J. Contemporary (1951–2001) evolution of lakes in the Old Crow basin, northern Yukon, Canada: remote sensing, numerical modeling, and stable isotope analysis. *Arctic* **62**, 225–238 (2009).
41. Necsoiu, M., Dinwiddie, C. L., Walter, G. R., Larsen, A., & Stothoff, S. A. Multitemporal image analysis of historical aerial photographs and recent satellite imagery reveals evolution of water body surface area and polygonal terrain morphology in Kobuk Valley National Park, Alaska. *Environ. Res. Lett*. **8**., doi.org/10.1088/1748-9326/8/2/025007 (2013).
42. Veremeeva, A. & Gubin, S. Modern tundra landscapes of the Kolyma Lowland and their evolution in the Holocene. *Permafrost Periglac.* **20**, 399–406 (2009).
43. Roach, J. K., Griffith, B., & Verbyla, D. Landscape influences on climate-related lake shrinkage at high latitudes. *Glob. Change Biol*. **19**, 2276–2284 (2013).
44. Rover, J., Ji, L., Wylie, B. K. & Tieszen, L. T. Establishing water body areal extent trends in interior Alaska from multi-temporal Landsat data, *Remote Sens. Lett.* **3**, 595-604 (2012).
45. Jepsen, S. M., Voss, C. I., Walvoord, M. A., Minsley, B. J., & Rover, J. Linkages between lake shrinkage/expansion and sublacustrine permafrost distribution determined from remote sensing of interior Alaska, USA. *Geophys. Res. Lett*. **40**, 882–887 (2013).
46. Sepulveda-Jauregui, A., Walter Anthony, K. M., Martinez-Cruz, K., Greene, S., & Thalasso, F. Methane and carbon dioxide emissions from 40 lakes along a north–south latitudinal transect in Alaska. *Biogeosci.* **12,** 3197-3223 (2015).
47. Walter Anthony, K. M. & Anthony, P. Constraining spatial variability of methane ebullition seeps in thermokarst lakes using point process models. *J. Geophys. Res. Biogeosci*., **118**, doi:10.1002/jgrg.20087 (2013).
48. Tan, Z., et al. Modeling CO_2 emissions from Arctic lakes: Model development and site-level study. *J. Adv. Mod. Earth Syst.*, 10.1002/2017MS001028 (2017).
49. van der Molen, M. K. et al. The growing season greenhouse gas balance of a continental tundra site in the Indigirka lowlands, NE Siberia. *Biogeosci.* **4**, 985-1003 (2007).
50. Jones, M. C., Grosse, G., Jones, B. M., & Walter Anthony, K. M**.** Peat accumulation in a thermokarst-affected landscape in continuous ice-rich permafrost, Seward Peninsula, Alaska. *J. Geophys. Res*. **117**, G00M07. doi:10.1029/2011JG001766 (2012).
51. Sturtevant, C. S. & Oechel, W. C. Spatial variation in landscape-level CO_2 and CH_4 fluxes from arctic coastal tundra: influence from vegetation, wetness, and the thaw lake cycle. *Glob. Change. Biol*. **19**, 2853-2866 (2013).
52. McGuire, A. D. An assessment of the carbon balance of Arctic tundra: comparisons among observations, process models, and atmospheric inversions. *Biogeosci*. **9**, 3185-3204 (2012).
53. Hayes, D. J. et al. Is the northern high-latitude land-based CO_2 sink weakening? *Glob. Biogeochem. Cyc*. **25**, doi:10.1029/2010GB003813 (2011).
54. Zhuang, Q. et al. Net emissions of CH_4 and CO_2 in Alaska: Implications for the region's greenhouse gas budget. *Ecolog. App*. **17**, 203-212 (2007).
55. Zona, D. et al. Characterization of the carbon fluxes of a vegetated drained lake basin chronosequence in the Alaskan Arctic Coastal Plain. *Glob. Change Biol*. **16**, 1870-1882 (2010).

56. Billings, W. D., & Peterson, K. M. Vegetational change and ice-wedge polygons through the thaw-lake cycle in arctic Alaska. *Arct. Alp. Res*. **12**, 413-432 (1980).
57. Olefeldt, D., Turetsky, M. R., Crill, P. M., & McGuire, D. A. Environmental and physical controls on northern terrestrial methane emissions across permafrost zones. *Glob. Change Biol*. doi:10.111/gbc.12071 (2012).
58. Regmi, P., Grosse, G. Jones, M. C., Jones,B. M., & Walter Anthony, K. Characterizing post-drainage succession in thermokarst lake basins on the Seward Peninsula, Alaska with TerraSAR-X backscatter and Landsat-based NDVI data. *Remote Sen.* **4**, 3741-3765 (2012).
59. van Huissteden, J. et al. Methane emissions from permafrost thaw lakes limited by lake drainage. *Nat. Clim. Change* **1**, 119–123 (2011).
60. Zona, D. et al. Cold season emissions dominate the Arctic tundra methane budget. *PNAS* **113**, 40-45 (2016).
61. Commane, R. et al. Carbon dioxide sources from Alaska driven by increasing early winter respiration from Arctic tundra. *PNAS*, doi/10.1073/pnas.1618567114 (2017).
62. Nicolsky, D. J., Romanovsky, V. E., Panda, S. K., Marchenko, S. S. & Muskett, R. R. Applicability of the ecosystem type approach to model permafrost dynamics across the Alaska North Slope. *J. Geophys. Res. Earth Surf.* **121**, doi:10.1002/2016JF003852 (2016).
63. Jorgenson, M. T., Shur, Y. L., & Pullman, E. R. Abrupt increase in permafrost degradation in Arctic Alaska. *Geophys. Res. Lett*. **33**, doi:10.1029/2005GL024960 (2006).
64. Raynolds, M. K. et al. Cumulative geoecological effects of 62 years of infrastructure and climate change in ice-rich permafrost landscapes, Prudhoe Bay Oilfield, Alaska. *Glob. Change Biol*. **20,** 1211-1224 (2014).
65. Grosse G., Jones B., & Arp C. Thermokarst Lakes, Drainage, and Drained Basins. In: John F. Shroder (ed.) Treatise on Geomorphology, Volume 8, pp. 325-353 (Academic Press, San Diego, 2013).
66. Kokelj, S. V. & Jorgenson, M. T. Advances in thermokarst research. *Permafr. Periglac. Process*. **24**, 108-119 (2013).
67. Arp et al. Threshold sensitivity of shallow Arctic lakes and sublake permafrost to changing winter climate. *Geophys. Res. Lett*. **43**, 6358–6365 (2016).
68. West, J. J. & Plug, L. J. Time-dependent morphology of thaw lakes and taliks in deep and shallow ground ice. *J. Geophys. Res*. **113**, F01009, doi:10.1029/2006JF000696 (2008).
69. Tan, Z., Zhuang, Q. & Walter Anthony, K. M. Modeling methane emissions from arctic lakes: model development and site-level study. *J. Adv. Model. Earth Sy*. **7,** doi:10.1002/2014MS000344 (2015).
70. Walter, K. M., Chanton, J. P., Chapin, F. S., Schuur, E. A. G., & Zimov, S. A. Methane production and bubble emissions from arctic lakes: Isotopic implications for source pathways and ages. *J. Geophys. Res. Biogeosci.* **113,** G00A08 (2008).
71. Lindgren, P. R., Grosse, G., Walter Anthony, K. M., & Meyer, F. Detection and spatiotemporal analysis of methane ebullition on thermokarst lake ice using high-resolution optical aerial imagery. *Biogeosci.* **13,** 27-44. (2016).
72. Frolking, S. & Roulet, N.T. Holocene radiative forcing impact of northern peatland carbon accumulation and methane emissions. *Glob. Change Biol.* **13**,1079–1088 (2007).
73. Joos, F. et al. Carbon dioxide and climate impulse response functions for the computation of greenhouse gas metrics: A multi-model analysis. *Atmos. Chem. Phys*. **13**, 2793–2825 (2013).
74. Archer, D, Kheshgi, H, & Maier-Reimer, E. Multiple timescales for neutralization of fossil fuel CO_2. *Geophys. Res. Lett*. **24**, 405-408 (1997).
75. Archer, D, Kheshgi, H, & Maier-Reimer, E. Dynamics of fossil fuel CO_2 neutralization by marine $CaCO_3$. *Glob. Biogeochem. Cyc.* **12**, 259-276 (1998).
76. Myhre, G. et al. In: *Climate Change 2013: The Physical Science Basis. Contribution of Working Group I to the Fifth Assessment Report of the Intergovernmental Panel on Climate Change* [Stocker, T.F., D. Qin, G.-K. Plattner, M. Tignor, S.K. Allen, J. Boschung, A. Nauels, Y. Xia, V. Bex and P.M. Midgley (eds.)]. Cambridge University Press, Cambridge, UK and New York, NY, USA (2013).
77. Denman, K. L. et al. Couplings Between Changes in the Climate System and Biogeochemistry. In: *Climate Change 2007: The Physical Science Basis*. Contribution of Working Group I to the Fourth Assessment Report of the Intergovernmental Panel on Climate Change [Solomon, S., D. Qin, M. Manning, Z. Chen, M. Marquis, K.B. Averyt, M.Tignor and H.L. Miller (eds.)]. Cambridge University Press, Cambridge, United Kingdom and New York, NY, USA (2007).
78. Mather, P. & Koch, M. Computer Processing of Remotely-sensed Images: An Introduction, Fourth ed. (John Wiley & Sons, Ltd, Chichester, 2011).

79. Blaschke, T. & Strobl, J. What's wrong with pixels? Some recent developments interfacing remote sensing and GIS. *GIS – Zeitschrift für Geoinformationssysteme* **14**, 12–17 (2001).
80. Navulur, K. Multispectral image analysis using the object-oriented paradigm. ISBN 9781420043068 (CRC Press, Inc., Boca Raton, 2007).
81. McFeeters, S. K. The use of the Normalized Difference Water index (NDWI) in the delineation of open water features. *Int. J. Rem. Sens*. **17**, 1425–1432 (1996).
82. Abraham, J. A promising tool for subsurface permafrost mapping: an application of airborne geophysics from the Yukon River Basin, Alaska. US Geol. Surv. Fact Sheet 2011–3133, 4 pp. (2011).
83. Minsley, B. J. et al. Airborne electromagnetic imaging of discontinuous permafrost. *Geophys. Res. Lett*. **39**, L02503, doi:10.1029/2011GL050079 (2012).
84. Jepsen, S. M., Voss, C. J., Walvoord, M. A., Minsley, B. J. & Rover, J. Linkages between lake shrinkage/expansion and sublacustrine permafrost distribution determined from remote sensing of Interior Alaska, USA. *Geophys. Res. Lett*. doi:10.1002/grl.50187 (2013).
85. Minsley, B. J., Wellman, T. P., Walvoord, M. A., & Revil, A. Sensitivity of airborne geophysical data to sublacustrine and near-surface permafrost thaw. *The Cryosphere* **9**, 781-794 (2015).
86. Emond, A. M. et al. Airborne electromagnetic and magnetic survey of the Goldstream Valley watershed, interior Alaska: Alaska Division of Geological & Geophysical Surveys Geophysical Report 2016-2. http://doi.org/10.14509/29681 (2017).
87. University of British Columbia's EM1DFM software: available at https://gif.eos.ubc.ca/software/em1dfm
88. Aarhus Workbench software: http://hgg.au.dk/software/aarhus-workbench/
89. GOCAD Software: http://www.pdgm.com/products/gocad/
90. Hoekstra, P. & McNeill, D. Electromagnetic probing of permafrost. Proc. 2nd Internat. Conf. On Permafrost. Nat. Acad. Sci., 517–526 (1973).
91. Olhoeft, G. R. 1978. Electrical properties of permafrost. Proc. 3rd Internat. Conf. On Permafrost. Nat. Res. Counc., Canada (1978).
92. Scott, W. J. & Kay, A. E. Earth resistivities of Canadian soils. Can. Elect. Assoc., Montreal. Vol 1, Main Rep., Vol 2., Site data (1988).
93. Vanhala, H., Lintinen, P., & Ojala, A. Electrical resistivity study of permafrost on Ridnitšohkka Fell in Northwest Lapland, Finland. *Geophysica* **45**, 103–118 (2009).
94. Pewe, T. L. Geology of the Fairbanks (D-2) Quandrangle, Alaska: U.S. Geological Survey Geologic Quadrangle Map 110, 1 sheet, scale 1:63, 360 (1958).
95. Department of Natural Resources, State of Alaska. Welts Map. http://dnr.alaska.gov/MapAK/browser?set=map&id=3793&extent={%22left%22:-195.72648437500288,%22bottom%22:47.009295368536456,%22right%22:-105.90226562500035,%22top%22:75.12915397505893,%22centerLonLat%22:null}
96. Nitze, I & Grosse, G. Detection of landscape dynamics in the Arctic Lena Delta with temporally dense Landsat time-series stacks. *Rem. Sens. Environ*. **181**, 27-41 (2016).
97. Breiman, L. Random forests. *Mach. Learn*. **45**, 5-32 (2001).
98. Strauss., J. et al. Database of ice-rich yedoma permafrost (IRYP). *PANGEA*, doi.org/10.1594/PANGAEA.861733 (2016).
99. Scandella, B. P., Varadharajan, C., Hemond, H. F., Ruppel, C. & Juanes, R. A conduit dilation model of methane venting from lake sediments. *Geophys. Res. Lett*. **38,** L06408 (2011).
100. Greene, S. et al. Modeling the impediment of methane ebullition bubbles by seasonal lake ice. *Biogeosci.* **11,** 6791-6811 (2014).
101. Walter Anthony, K. M. et al. Estimating methane emissions from northern lakes using ice bubble surveys. *Limnol. Oceanogr. Meth.* **8**, 592–609 (2010).
102. Zimov, S. A. et al. Flux of methane from north Siberian aquatic systems: Influence on atmospheric methane, in: Permafrost Response on Economic Development, Environmental Security and Natural Resources, NATO Science Series 2, 76, edited by: Paepe, R. and Melnikov, V. P. pp. 511–524 (Kluwer Academic Publishers, Dordrecht, Netherlands and Boston, Massachusetts, USA, 2001).
103. Walter Anthony, K. M., Anthony, P., Grosse, G., & Chanton, J. Geologic methane seeps along boundaries of arctic permafrost thaw and melting glaciers. *Nat. Geosci.* **5**, 419-426 (2012).

104. Chanton, J. P. et al. Radiocarbon evidence for the importance of surface vegetation on fermentation and methanogenesis in contrasting types of boreal peatlands. *Global Biogeochem. Cy*, **22**, GB4022 (2008).
105. Vogel, J. S., Turteltaub, K. W., Finkel R. & Nelson, D. E. Accelerator mass spectrometry. *Anal. Chem*. **67**, 353A-359A (1995).
