# Supplementary material for: 21st-century modeled permafrost carbon emissions accelerated by abrupt thaw beneath lakes
Source: Nat Commun. 2018 Aug 15;9:3262. doi: 10.1038/s41467-018-05738-9 (PMC6093858; doi:10.1038/s41467-018-05738-9)
Supplement: Supplementary file 3 — Description of Additional Supplementary Files [file 41467_2018_5738_MOESM3_ESM.pdf]

Description of Additional Supplementary Files

File Name: Supplementary Data 1

Description: AThaw and CLM4.5BGC model emissions (annual and cumulative since 2010) and associated radiative forcing (RF). Model results under Representative Concentration Pathway (RCP) 4.5 and RCP8.5 climate scenarios are shown in *a* and *b*, respectively. AThaw emissions are the median and 68% uncertainty range (in parentheses) from a 500-member AThaw model ensemble, which considers uncertainty in 18 key model parameters containing critical factors that influence AThaw modeled emissions.

File Name: Supplementary Data 2

Description: Radiocarbon dates of CH_4 and CO_2 in thermokarst lake bubbles. ^{14}C-depleted CH_4 values correspond to ^{14}C-depleted permafrost soil organic carbon values in the study lake regions[21]. The $\delta^{13}C$ of CH_4 ranged from -55‰ to -82‰ (mean ± standard deviation: -69‰ ± 5‰), indicating a microbial methane source.
